# Modeling tau spreading in Alzheimer’s disease in the AD workbench environment

**DOI:** 10.1002/alz.095826

**Published:** 2025-01-09

**Authors:** Nicolai Franzmeier, Michael Schöll

**Affiliations:** ^1^ Institute for Stroke and Dementia Research, Ludwig‐Maximilians‐Universität München, LMU München, Munich Germany; ^2^ Department of Psychiatry and Neurochemistry, Institute of Neuroscience and Physiology, The Sahlgrenska Academy, University of Gothenburg, Mölndal Sweden

## Abstract

**Background:**

To understand the progression of Alzheimer's disease (AD), neuroimaging and biomarker research relies increasingly on sophisticated data analysis techniques that are often restricted to expert lab environments. Here, we demonstrate how complex analyses on modeling tau spreading across interconnected brain regions from our previous studies (e.g., Franzmeier et al., Nat Commun, 2020; 2024, Sci Adv, 2020) can be replicated using the cloud‐based Alzheimer's Disease Workbench (ADWB) environment. We illustrate how data analyses can transition from restricted lab environments to open cloud‐based workspaces, highlighting the potential for data sharing, transparency and collaborative analysis in AD research via cloud‐based solutions.

**Methods:**

Data from the Alzheimer's Disease Neuroimaging Initiative (ADNI) were re‐analyzed in the ADWB environment. The analyses involved combining resting‐state fMRI with longitudinal tau‐PET imaging data to investigate the relationship between brain connectivity and tau accumulation. We illustrate the role of the ADWB as an integrated workspace where datasets can be uploaded, explored, and analyzed using a suite of tools, including Python and R. Within this environment we illustrate how sophisticated data analyses can be replicated in shared environments to facilitate collaborative research efforts.

**Results:**

Replicating key findings from our original studies, we demonstrate that interconnected brain regions show correlated tau accumulation, and that tau spreads from local epicenters across connected brain regions, indicating potential trans‐neuronal tau propagation. We provide shared data and code for running and visualizing analyses on connectivity‐mediated tau spreading, enhancing the accessibility and reproducibility of neuroimaging analyses in AD.

**Conclusion:**

The AD Workbench environment effectively supports complex neuroimaging analyses, facilitating data sharing and collaborative research using open‐access datasets such as ADNI. By enabling researchers to replicate and extend existing studies, ADWB can enhance our understanding of AD mechanisms and foster collaborations across different research sites and groups. We underscore the role of cloud‐based and open‐access solutions in neuroimaging research that can make advanced data analysis techniques available to a broader research community.

